# Hybridizing machine learning in survival analysis of cardiac PET/CT imaging

**DOI:** 10.1007/s12350-023-03359-4

**Published:** 2023-09-01

**Authors:** Luis Eduardo Juarez-Orozco, Mikael Niemi, Ming Wai Yeung, Jan Walter Benjamins, Teemu Maaniitty, Jarmo Teuho, Antti Saraste, Juhani Knuuti, Pim van der Harst, Riku Klén

**Affiliations:** 1 Department of Cardiology, Division Heart & Lungs, University Medical Center Utrecht, Utrecht University, Utrecht, The Netherlands; 2 Department of Cardiology, University of Groningen, University Medical Center Groningen, Groningen, The Netherlands; 3 Turku PET Centre, University of Turku and Turku University Hospital, Kiinamyllynkatu 4-8, 20520 Turku, Finland; 4 Heart Center, Turku University Hospital, Turku, Finland

**Keywords:** Machine learning, survival analysis, cardiovascular events, hybrid imaging, PET/CT

## Abstract

**Background:**

Machine Learning (ML) allows integration of the numerous variables delivered by cardiac PET/CT, while traditional survival analysis can provide explainable prognostic estimates from a restricted number of input variables. We implemented a *hybrid* ML-and-survival analysis of multimodal PET/CT data to identify patients who developed myocardial infarction (MI) or death in long-term follow up.

**Methods:**

Data from 739 intermediate risk patients who underwent coronary CT and selectively stress ^15^O-water-PET perfusion were analyzed for the occurrence of MI and all-cause mortality. Images were evaluated segmentally for atherosclerosis and absolute myocardial perfusion through 75 variables that were integrated through ML into an ML-CCTA and an ML-PET score. These scores were then modeled along with clinical variables through Cox regression. This *hybridized* model was compared against an expert interpretation-based and a calcium score-based model.

**Results:**

Compared with expert- and calcium score-based models, the hybridized ML-survival model showed the highest performance (CI .81 vs .71 and .64). The strongest predictor for outcomes was the ML-CCTA score.

**Conclusion:**

Prognostic modeling of PET/CT data for the long-term occurrence of adverse events may be improved through ML imaging score integration and subsequent traditional survival analysis with clinical variables. This *hybridization* of methods offers an alternative to traditional survival modeling of conventional expert image scoring and interpretation.

**Supplementary Information:**

The online version contains supplementary material available at 10.1007/s12350-023-03359-4.

## Introduction

Worldwide, coronary artery disease (CAD) remains the most common cause of death, but our ability to accurately diagnose significant CAD through advanced non-invasive modalities has greatly improved in the last decades. However, there is a pressing necessity to optimize *prognostic* estimations at the individual-level for the risk of developing adverse cardiovascular outcomes.^1–3^ Performance of clinical risk scores and single imaging variables (such as coronary Calcium Score) has been modest in this challenging task, and it is hypothesized that large imaging datasets might contain insights that can improve the identification of patients at risk.

Coronary computed tomography angiography (CCTA) and positron emission tomography (PET) represent state-of-the-art techniques that allow accurate evaluation of coronary anatomy and absolute quantitative myocardial perfusion, respectively. CCTA provides luminal spatial visualization of the coronary arteries through contrast images that are segmentally analyzed for the presence of atherosclerotic plaque, luminal narrowing, and calcification as recommended in the SCCT guidelines.^4^ On the other hand, PET imaging allows the quantitative evaluation of myocardial blood flow (in mL·min^−1^·g^−1^ of tissue) throughout 17 left ventricular segments (as recommended by the American Heart Association) in order to objectify myocardial ischemia. As such, advanced cardiac imaging with PET/CCTA delivers a large number of variables describing the *anatomic-functional* state of the heart that carry intrinsic complex interrelations that may not be fully characterized by traditional statistical modeling and will vary between individuals.

Nowadays, machine learning (ML) has rapidly stoked a revolution in medical data analysis. ML algorithms are able to explore and *integrate* large numbers of variables (through complex non-linear dependencies) in order to boost performance in classification and prediction tasks.^5^ The implementation of ML in cardiovascular imaging has already delivered exciting proofs of concept in the evaluation of a number of pathological conditions including CAD.^6–10^

Notably, the utility of ML in predicting clinical outcomes such as mortality, revascularization, heart failure and myocardial infarction (MI) has been recently explored in both CCTA^6^ and PET^11^ data independently. However, there is a paucity of imaging studies regarding the implementation of ML for prognostic analysis of cardiovascular outcomes considering the influence of individual time-to-event^12,13^ (e.g., through survival modeling through Cox proportional hazards).

Hence, given that advanced PET/CCTA imaging delivers a large number of interrelated variables suitable for ML integration and that *survival* analysis could be utilized on the output from ML methods in cardiovascular imaging, the present study sought to develop and evaluate the performance of a hybridized ML-and-survival analysis workflow for cardiac PET/CCTA and clinical data in the long-term prediction of MI and death at the individual level.^14,15^ We additionally compared this workflow’s performance to that of prior published statistical models and of Calcium Score as a powerful imaging-derived predictor of adverse outcomes.

## Methods

### Study population

We retrospectively evaluated 951 consecutive symptomatic patients with intermediate pre-test probability of CAD (based on the pre-test probability categorization in line with the 2019 ESC CCS guidelines) who had been referred to CCTA in the PET Centre of the Turku University Hospital in Finland. According to the local sequential imaging protocol, patients with suspected obstructive stenosis on CCTA underwent downstream stress PET perfusion imaging to evaluate the hemodynamic significance of stenosis.^3^ Patients with documented CAD, prior MI, or prior revascularization (PCI or CABG) were not included in the present study. After exclusion of patients with non-diagnostic imaging results or failure to adhere to the local sequential imaging protocol, data from 739 subjects were analyzed. A report of CCTA and PET findings by cardiovascular imaging specialist, as recommended by SCCT guidelines, was provided to the treating physician to guide patient management. The study was performed in accordance with the Declaration of Helsinki. The Ethics Committee of the Hospital District of Southwest Finland waived the need for written informed consent owing to retrospective observational study design.

### Clinical variables

Demographic (sex and age) and clinical data (hypertension, dyslipidemia, smoking status, type 2 diabetes mellitus, family history of cardiovascular disease, chest complaints, and dyspnea) were extracted from the electronic medical records system and are summarized in Table 1.

**Table 1:** Characteristics of baseline risk factors and imaging expert interpretations

Variables	Training set n = 493	Independent test set n = 246	*p* value
*Demographics*			
Male sex (%)	219 (44.4)	103 (41.9)	.562
Age (years)	61.6 (9.5)	60.1 (9.1)	.691
*Risk factors*			
Smoking status			.875
No	331 (67.1)	170 (69.1)	
Previously	103 (20.9)	38 (15.4)	
Currently	59 (12.0)	38 (15.4)	
Type 2 Diabetes			.340
No	336 (68.2)	177 (72.0)	
Pre-diabetes	85 (17.2)	35 (14.2)	
Yes	72 (14.6)	34 (13.8)	
Hypertension	261 (52.9)	135 (54.9)	.675
Dyslipidemia	307 (62.2)	144 (58.5)	.368
*Imaging findings*			
Obstructive CAD in CCTA (%)	235 (47.7)	125 (42.4)	.304
Abnormal myocardial perfusion (%)	123 (24.9)	71 (24.1)	.112
Family history (%)	216 (43.8)	112 (45.5)	.510
Chest complaints (%)	352 (71.4)	200 (81.3)	.004

### Follow-up and clinical outcomes

The analyzed endpoints were recorded in binary form for the occurrence of MI or all-cause death using the registries of the Finnish National Institute for Health and Welfare and the Centre for Clinical Informatics of the Turku University Hospital. Identified events were manually confirmed by the investigators through the electronic medical records system following the European Society of Cardiology recommendations. There were no missing data in this regard for our sample.

### Imaging acquisition

Patients underwent sequential imaging protocol with CCTA and selective ^15^O-water stress PET perfusion imaging. The corresponding acquisition protocols have been described previously.^1^ CCTA scans were performed in a 64-row PET/CT scanner (GE Discovery VCT or GE D690, General Electric Medical Systems, Waukesha, Wisconsin). Prior to acquisition, 0-30 mg of metoprolol were administered intravenously to achieve a target heart rate of <60 bpm and 1.25 mg of isosorbide dinitrate aerosol, or alternatively 800 mg of sublingual nitrate, were also administered. CCTA utilized intravenously administered low-osmolal iodine contrast agent (48-155 mL at 320-400 mg iodine·mL^−1^) and prospective ECG-triggered acquisition.

Dynamic quantitative PET myocardial perfusion imaging during pharmacological stress (using adenosine infusion of 140 µg·kg^−1^·min^−1^ as vasodilator) was performed as previously described.^1^ The mean injected activity of ^15^O-water was 1042 ± 117 MBq. All patients were instructed to refrain from methyl-xanthine-containing food, beverages, and medications (e.g., coffee, chocolate, tea) for 24 hours before the PET study.

### Image analysis

CCTA data was analyzed according to the segmentation system recommended in the SCCT guidelines.^4^ In detail, the coronary artery tree was described by: (1) its system dominance [right, left or co-dominance], (2) the anatomical presence or absence of each theoretical coronary segment, (3) the presence or absence of atherosclerotic plaque per segment, (4) the visually estimated percentage of luminal narrowing [0%, < 50%, 50-69%, 70-99% or 100%], and (5) the complete, partial or absent calcification of an atherosclerotic plaque when present. The database was coded in such a way that it allowed for the distinction between normal coronary segments and anatomically absent ones (0 and 1 values, respectively).

From these recorded variables, we implemented the statistical CCTA score proposed by De Graaf et al., which is generated by calculating plaque-, stenosis- and segment weight factors for each coronary segment and summing individual segment scores together as described previously.^16^ The de Graaf score is based on linear integration of these variables as an example comparator of a non-ML based approach to CCTA scoring.

Coronary Calcium Score^17^ by the Agatston method was globally quantified and stored as a unique continuous variable.

PET data were quantitatively analyzed using Carimas software (Turku PET Centre, Finland) using a one-tissue (two-compartment) kinetic model to estimate absolute stress myocardial blood flow (MBF) through the standardized 17-segment AHA model (where segments 2 and 3 being negated due to correspondence to the membranous interventricular septum).^2^ The finding of at least one segment with a stress MBF of < 2.4 mL·g^−1^·min^−1^ was considered abnormal and indicative of myocardial ischemia.^3^ Missing values were considered as null parameters and input accordingly.

In total, this segmental anatomo-functional description delivered 58 CCTA anatomically descriptive variables, a single continuous Calcium Score variable, and 15 PET variables. All image interpretations were performed by an experienced cardiovascular imaging physician and recorded in a standardized reporting system.

### Machine learning

**Figure 1:**
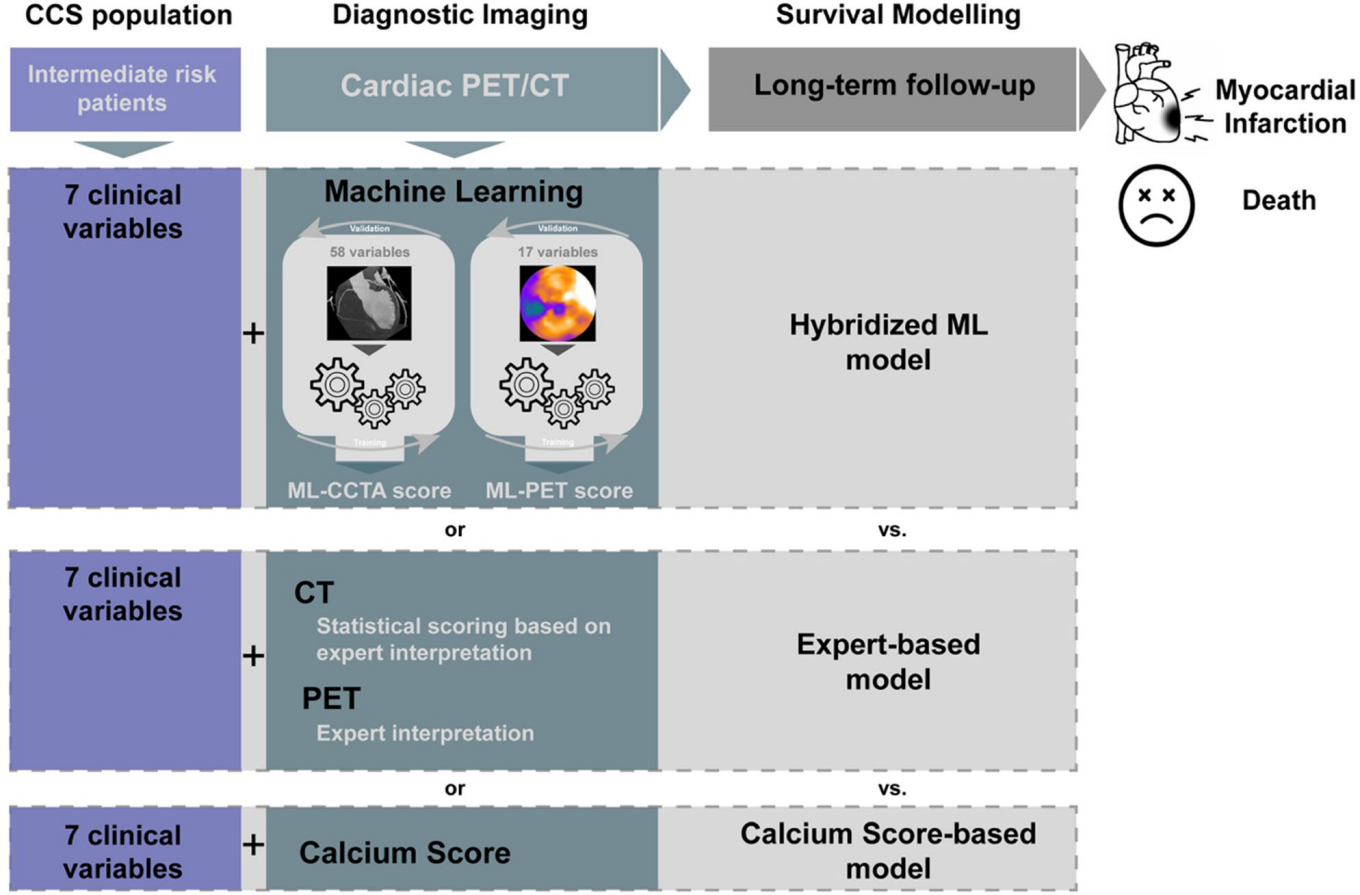
Machine learning and survival workflow. *CCS*, chronic coronary syndromes; *CCTA*, coronary computed tomography angiography; *ML*, machine learning; *PET*, positron emission tomography

Our machine learning workflow (Figure 1) was generated in accordance with current state-of-the-art recommendations and the Proposed Requirements for Cardiovascular Imaging-Related Machine Learning Evaluation (PRIME) Checklist,^18^ which can be consulted in the supplement.

#### Data pre-processing and re-sampling

Missing values in in clinical data were imputed with negative value for smoking (N = 91 in training and N = 28 in test dataset), diabetes (N = 116 in training and N = 47 in test dataset), hypertension (N = 80 in training and N = 43 in test dataset), and dyslipidemia (N = 85 in training and N = 40 in test dataset). The variables gender and age did not have missing values. Imaging variables did not have missing values except in the case the subject did not proceed to PET scan. In these cases, all PET variables (N = 259 in training and N = 126 in test dataset) were assigned to zero. ML analytics utilized gradient boosting machine (GBM)^5^ algorithm for the analysis based on robustness and stability. The 739 subjects were randomly assigned to a training or testing dataset (with a 2:1 split ratio to achieve datasets with N = 493 and N = 246).^19,20^ A 10-fold cross-validation policy was applied to tune model parameters through the ML training and validation process for the training dataset (N = 493). Model building and optimization were exclusively performed in the training dataset, while final comparative performance evaluations were conducted in the test dataset. Feature selection considering the most informative CCTA and PET variables was performed in model building as a recommended measure to promote dimensionality reduction.

#### ML-integrated scores

ML modeling was performed as aforementioned separately in CCTA (58) and PET (17) variables. The outcome for both ML models was MI or all-cause death. The resulting continuous output (a pseudo-probability expressed in a range from 0 to 1) integrated the extensive imaging information into a single ML-CCTA score and a single ML-PET score. These ML-integrated imaging scores were further utilized in the subsequent survival analysis (see below).

### (Hybridized) survival analysis

Continuous variables are presented as mean and standard deviation (SD). Categorical variables were expressed as counts and corresponding percentages. Significant statistical differences in baseline characteristics between the training and testing datasets were compared using the Wilcoxon rank sum test for continuous variables and the chi-squared test for categorical ones.

The isolated performance of the integrated ML scores (ML-CCTA and ML-PET) was evaluated through the area under the curve (AUC) obtained from receiver operating characteristic (ROC) analyses.

The prognostic analyses were performed through Cox regression modeling^21^ for the occurrence of the previously described composite endpoint (i.e., MI or all-cause death). Predictive performance in test data was illustrated using Kaplan-Meier curves accompanied with log-rank test *P* values. The resulting *hazard ratios* were expressed along with their 95% confidence intervals.

The independent and comparative significance of the ML-integrated scores was evaluated through the following three predictive models, namely: (1) *Hybridized ML Model:* considering clinical variables, ML-CCTA score and ML-PET score, (2) *Expert-based Model:* considering clinical variables, CCTA expert interpretation and PET expert interpretation, and (3) *Calcium score-based Model:* considering global Calcium Score. Performance was evaluated through the *concordance index* (CI) by binary transformation of its predictions to a binary Youden’s J statistic applied to training dataset.^22^ Concordance indexes were compared using Student’s *t* test.^23,24^ A two-tailed *P* value < .05 was considered statistically significant.

Statistical and Machine Learning analytics were implemented using R (version 3.5.3) with complementary sub-packages *survival* (version 2.44-1.1), *survcomp* (version 1.36.1) and *gbm* (version 2.1.5) for GBM.

## Results

### Study population and imaging findings

Baseline characteristics of the study population are described in Table 1*.* There were no statistically significant differences between the training and test datasets. The population consisted 55.6% and 58.9% of females, and the mean age was 61.6 ± 9.5 and 60.1 ± 9.1 in the training and test samples, respectively. In general, a majority of patients were not smokers, while a substantial proportion had arterial hypertension and dyslipidemia. In training data 48% (n = 235) of the patients that underwent CCTA were shown to have anatomically suspected obstructive CAD, and subsequently, underwent stress PET perfusion imaging. In test data this proportion was 42% (n = 125). Approximately half of those who underwent downstream PET imaging had abnormal myocardial perfusion with a median of 7 affected myocardial segments (IQR 2-12) in the training data. Median was 5 for the test data (IQR 2-12).

### Outcomes

Median follow-up was 6.1 years (IQR 5.4-7.4) for the training data and 6.0 years (IQR 5.3-7.5) for the test data. Endpoint (all cause death or MI) cases (46 events) were distributed as follows: 33/493 (6.7%) in the training data and 13/246 (5.3%) in the test data.

### Machine learning scores integration

The generated ML-CCTA score integrated 28 variables of the 58 initial variables selected through GBM feature selection, which is shown in Table S1. This score showed a univariable concordance index of .70 and an AUROC of .72 in the test data. More details about CCTA variable characteristics are presented in Table S3.

The generated ML-PET score integrated all segmental perfusion variables (15) and documented a concordance index of .58 and an AUROC of .63. More details about PET variable characteristics are presented in Table S4 and relative variable influence to ML-PET score are presented in Table S2.

### Survival analyses

**Figure 2:**
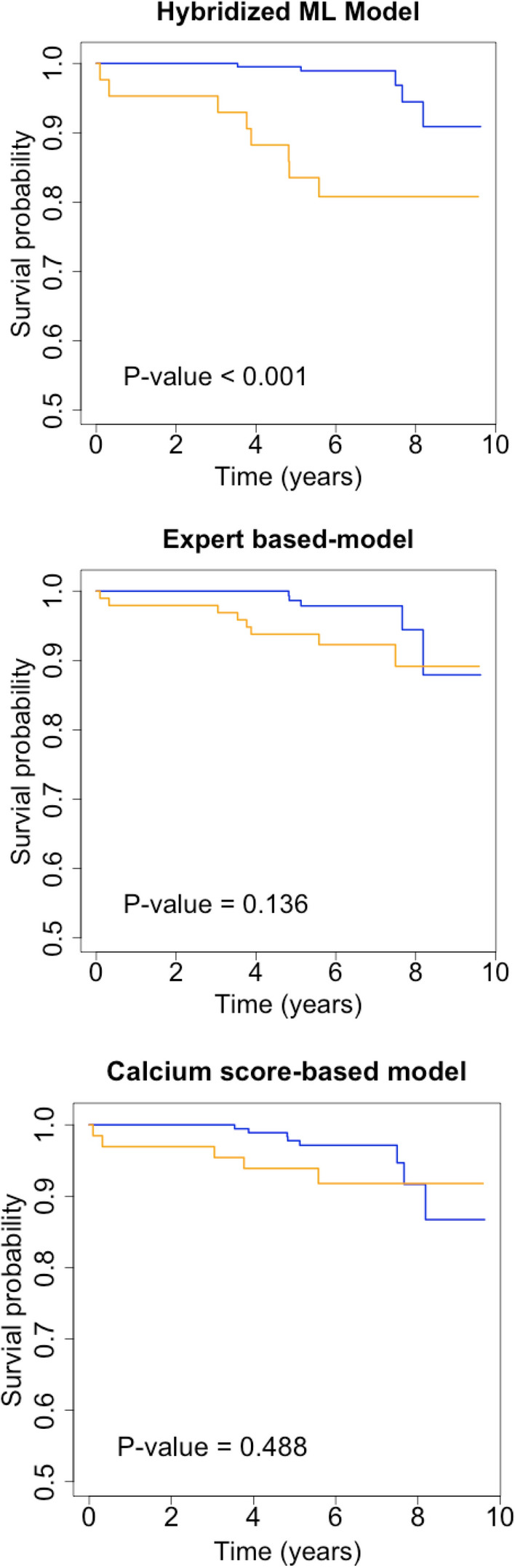
Kaplan–Meier curves of the hybridized ML model and two reference models. Blue curve corresponds to low probability of outcome and orange to high probability of outcome

Both concordance index and AUROC values for the final hybridized ML model were higher than the reference methods. Based on the Kaplan-Meier curves our *hybridized ML model* was able to significantly discriminate the occurrence of event between low- and high-risk patient groups (as depicted in Figure 2), beyond the performance of the alternative models namely, the *expert-based model* and the *calcium score-based model* (Figure 2).

Cox regression analysis for Hybridized ML Model showed only ML-CCTA score (Hazard ratio 15.26; 95% CI 5.38-43.25; *P* value, < .0001) and ML-PET score (Hazard ratio 4.23; 95% CI 1.99-8.98; *P* value, .0002) were significant endpoint predictors. Only significant predictive variable for Expert-based Model was the statistical CCTA score (Hazard ratio 1.10; 95% CI 1.04-1.15; *P* value, .0006). Calcium score-based Model consisted of only one variable, Calcium Score (Hazard ratio, 1.16; 95% CI 1.03-1.30; *P* value, .0012), which was significantly associated with endpoints. Overall CCTA derived variables (ML-CCTA score, statistical score, and Calcium Score) were independently associated with endpoints throughout the three models. The results of Cox regression analysis are presented in Table 2.

**Table 2:** Survival models through Cox proportional hazards utilizing clinical variables, ML-derived scores and comparator predictors (CCTA statistical score by De Graaf and Calcium Score)

Predictors	HR	95% CI	*P* value
Hybridized ML model			
Age	1.02	.97–1.06	.360
Smoking	.95	.41–2.18	.897
Diabetes	1.06	.69–1.62	.772
Hypertension	1.03	.48–2.18	.934
Dyslipidemia	.71	.33–1.50	.376
ML-CCTA	15.26	5.38–43.25	< .0001
ML-PET	4.23	1.99–8.98	.0002
Expert-based model			
Age	1.03	.99–1.08	.129
Smoking	1.48	.69–3.16	.311
Diabetes	1.33	.86–2.06	.195
HTN	1.12	.51–2.40	.775
DLP	.71	.33–1.53	.388
Statistical CCTA score	1.10	1.04–1.15	.0006
Clinical PET	1.02	.58–1.77	.947
Calcium score-based model			
Calcium score	1.16	1.03–1.30	.0012

*CI*, confidence interval; *HR*, hazard ratio

Adjusted Cox regression analyses demonstrated that the Hybridized ML Model (i.e., that including clinical variables, the ML-CCTA and ML-PET scores) had the best predictive performance for the occurrence of the studied outcomes with a concordance index of .85 for training and .81 for the test data, while the corresponding AUROC values were .89 and .76, respectively. Alternatively, the Expert Based Model (i.e., utilizing clinical variables and the expert interpretations of CCTA [through the statistical score by DeGraaf et al.] and of PET) documented an intermediate performance with a concordance index of .72 for training and .71 for the test dataset, and AUROC values of .76 and .67, respectively. Finally, the Calcium score-based Model showed discrete performance with a concordance index of .65 for training and .64 for the test dataset and AUROC values of .68 and .64, respectively. These results along with 95% CIs are shown in Table 3. Although the specified significance threshold was not reached, there was a clear trend toward an improved predictive discrimination of the *hybridized ML model* with respect to the *expert-based model* in the test data (Concordance index, *P* value = .076; AUROC, *P* value = .731), while a significant difference was patent between the *hybridized ML model* and the *calcium score-based model* (Concordance index, *P* value = .014; AUROC, *P* value = .025).

**Table 3:** Concordance index (C-index) and area under receiver operating characteristic curve (AUROC) for the hybridized ML model and reference models, confidence interval inside paranthesis

	Hybridized ML model		Expert-based model		Calcium score-based model	
	Training	Testing	Training	Testing	Training	Testing
C-index	.85 (.75–.95)	.81 (.67–.95)	.72 (.61–.84)	.71 (.55–.87)	.65 (.54–.77)	.64 (.49–.79)
AUROC	.89 (.81–.97)	.76 (.63–.93)	.76 (.66–.86)	.67 (.50–.84)	.68 (.59–.78)	.64 (.49–.79)

## Discussion

The present study describes the implementation of ML in the integration of CCTA and PET myocardial perfusion variables into single scores (ML-CCTA and ML-PET, respectively), which subsequently showed to be significant survival predictors (in an adjusted Cox proportional hazards analysis) for the occurrence of MI and all-cause mortality considering a long-term follow-up registry of patients at intermediate risk of CAD. It proposes method *hybridization* involving ML (gradient boosting machine) and traditional statistical methods (Cox regression) for the analysis of cardiac imaging data. This workflow proposes the value of harnessing complementary ML and traditional analyses in prognostic studies in cardiovascular imaging research.

Currently, advanced cardiovascular imaging delivers a myriad of variables that characterize the (patho)physiological profile of the heart both anatomically and functionally. This complementary nature is a known advantage of hybrid imaging with SPECT/CT or PET/CT in coronary artery disease.^25^ However, the amount of data even from a single imaging modality represents a challenge for traditional statistical analytics commonly employed in clinical research studies as they tend to not converge given to many input variables and few relevant outcomes. Nowadays, evaluation of these data is performed by an expert clinician who consolidates the information into a categorical summary (interpretation) of the extent or severity of the disease (through what may be called *natural intelligence*). Alternative approaches developed are the generation of statistical scores that combine variables through linear dependencies and the extraction of single biomarkers with population-based prognostic value (such as coronary Calcium Score).^26^ The effective operators of these premises are embodied in the present study, firstly in the resulting 58 variables from CCTA (based on dominance, segmental presence of plaque, degree of luminal narrowing and calcification) and the 17 variables from PET imaging (segmental absolute myocardial blood flow during stress), and secondly in the selected comparator predictors (the reference CCTA score promoted by De Graaf and colleagues and the global Calcium Score) analyzed.

ML analytics allow the integration of numerous interrelated variables^6^ through the exploration of complex non-linear dependencies between them. This enhances their value in classification tasks. In this study, ML facilitated imaging feature integration through a cross-validated workflow that delivered an ML-CCTA score and an ML-PET score. These ML-based single number scores aggregate the value of the extensive information provided by each imaging technique, which a demonstrated strength of ML. Then, these ML-derived scores demonstrated to be strong univariable and multivariable predictors (in the strongest of definitions, which considers time-to-event modeling) of infarction and mortality through *traditional* Cox regression modeling (with adjusted HR of 15.3 and 4.2 for the ML-CCTA and ML-PET scores, respectively); clearly overshadowing the coefficients of the utilized statistical CCTA score (De Graaf et al.) and the expert PET interpretation variable in the comparator model. The prognostic coefficients of such ML-based scores also were superior to that of the model utilizing coronary Calcium Score, which in itself represents a valued prognostic predictor for incident cardiovascular disease and adverse events in already suspected CAD^26^ documented in large populational studies. Our findings suggest that the generation of scores through ML that integrate all the resulting variables from advanced cardiovascular imaging (with PET/CT) may boost its prognostic utility beyond the current boundaries of clinical interpretation and single biomarkers. Whether this possibility may translate into improved cardiovascular outcomes through better patient selection for treatment individualization remains unknown and will require tailored prospective studies.

Lately, it is suggested that, in spite of the increasing reports supporting the improved performance of ML algorithms in classification tasks over conventional statistical methods, the value of traditional analytics should not be disregarded as it may be comparable and even preferable in some instances.^27^ We believe a sober consideration of the potential analytical advantages of ML and rather conventional statistics is continuously warranted. Therefore, this study harnessed and engaged the advantages of *traditional* survival modeling through Cox regression such as interpretability (explainability), time-to-event consideration, and robustness of its well-known estimates (i.e., hazard ratios), as well as their familiarity to both clinicians and clinical researchers, while also engaging the advantage of multiple variable integration through ML analytics. Recently, this issue has also been nicely investigated by Pieszko et al.^12^ suggesting the inherent need for better prognostic estimates by harnessing the capacities of ML. Here, our approach was selected after considering that full ML survival modeling requires larger amounts of data and that traditional Cox regressions may not easily handle a large number of predictors in the long term (e.g., all the imaging variables) for the given amount of events recorded.

In this report, we could not unequivocally prove the superiority of the proposed *hybridization* of methods. However, we documented how it might render an optimized approach to explainable data handling through machine learning for advanced cardiovascular imaging. A major feature of this vision is that its implementation is certainly not restrictive to PET/CT imaging as our proposed methods are expandable to any sort of non-invasive or invasive imaging as well as to any realm where large amounts of complex interrelated prospective data is readily available.

Presently, risk prediction based on a prognostic workflow involving ML-based scores may be both useful when considering negative and positive cases although the risk of misclassification should be differently weighted. In this sense, we envision an initial implementation in which negatively predicted cases may be ruled-out so that clinical attention can be focused on positively predicted cases in which a more nuanced clinical evaluation may be required. This initial approach may promote (human) resource economization while minimizing the potential risk of depositing direct responsibility of positive case identification on a tailored experimental model. Currently, optimal assessment of event risk prediction in patients with CAD is a clear gap in knowledge as stated in the recent 2019 ESC CCS guidelines.

## New knowledge gained

The generation of ML-based imaging scores from coronary CT and PET perfusion data is feasible and delivers significant prognostic predictors for the development of myocardial infarction and death. Traditional survival analysis of ML-based scores offers an interpretable approach that may better exploit the potential of wide imaging data.

## Limitations

The present study naturally carries all the intrinsic disadvantages of an observational study even if longitudinal. Unfortunately, this evaluation was not extended to an external (validation) cohort yet as the nature of the present registry is unique among advanced imaging centers (selective PET/CT with ^15^O-water and long-term follow-up). Nevertheless, our objective was mainly to explore for the first time the feasibility of hybridizing a ML and Cox regression approach in order to perform a prognostic evaluation of advanced cardiac imaging data. Further evaluation of external generalizability will follow in due course. Another limitation may be the low number of outcomes (6.2%) which may have reduced the predictive power of the models. Yet, such rate was comparable to other long-term observational studies dealing with hard cardiovascular outcomes and results therefore representative. Of note, the setup of this long-term registry implies the selective performance of PET scanning on patients with possibly obstructive CAD in the initial CT imaging. As such, roughly half of the patients analyzed only had CT data as PET imaging was not performed. This might negatively influence the obtained c-index. Hence, performance might have been underestimated.

We also recognize that determining the clinical value of such approach in a clinical scenario is warranted and not yet performed.

## Conclusion

Prognostic modeling of cardiac PET/CT data for the long-term occurrence of MI and all-cause mortality may be improved through integration of imaging data into ML-based scores and subsequent survival analysis along with clinical variables. This *hybridization* of methods engages novel ML analytics in a more familiar way for clinicians, while offering an alternative to traditional survival modeling of conventional image interpretation. The combination of ML and conventional statistics may boost the prognostic value of cardiac imaging at the individual level and warrants further research.

### Supplementary Information

Below is the link to the electronic supplementary material.

Supplementary file1 (DOCX 31 kb)

Supplementary file2 (PPTX 1170 kb)

## Abbreviations

AUROC: Area under the receiver operating characteristic curve
CAD: Coronary artery disease
CABG: Coronary artery bypass graft
CCTA: Coronary computed tomography angiography
GBM: Gradient boosting machine
HR: Hazard ratio
MBF: Myocardial blood flow
MI: Myocardial infarction
ML: Machine learning
PCI: Percutaneous coronary intervention
PET/CT: Positron emission tomography/computed tomography
