# Supplementary material for: Hybridizing machine learning in survival analysis of cardiac PET/CT imaging
Source: J Nucl Cardiol. 2023 Sep 1;30(6):2750–9. doi: 10.1007/s12350-023-03359-4 (PMC10682215; doi:10.1007/s12350-023-03359-4)
Supplement: Supplementary file 1 — Supplementary file1 (DOCX 31 kb) [file 12350_2023_3359_MOESM1_ESM.docx]

**SUPPLEMENT**

In this Supplement we represent details of the used data, analysis and compared ML methods including concordance index, AUROC, sensitivity and specificity results.

Checklist for standardized reporting of Machine Learning investigations.

1. Designing the Study Plan
   1. Describe the need for the application of machine learning to the dataset

*The large number of interrelated variables resulting from CCTA and PET imaging and their untapped integrative value in this specific prediction problem warrants the implementation of ML.*

- 1. Describe the objectives of the machine learning analysis
     1. Define the study plan

*Defined in the Methods section (input and output variables through cross-validated feature selection and ML modelling).*

- - 1. Describe the summary statistics of baseline data

*Described in Table 1.*

- - 1. Describe the overall steps of the machine learning workflow

*Described in Methods and Supplement.*

1. Data Standardization, Feature Engineering, and Learning
   1. Describe how the data were processed in order to make it clean, uniform, and consistent
      1. Describe whether variables were normalized and if so, how this was done

*Described under Methods. No normalization was used in data processing*

- - 1. Provide details on the fraction of missing values (if any) and imputation methods

*As described in Methods. Missing values in clinical variables were replaced with zero corresponding to no disease as stated in methods. Missing value fractions are listed in Supplement S5.*

- - 1. Describe any feature selection processes applied

*Described in the Methods. Only feature selection was applied in the modelling process. All imaging variables were analyzed.*

- - 1. Identify and describe the process to handle outliers if any

*Outliers were no excluded from the analysis. Extreme outliers were not present.*

- - 1. Describe whether class imbalance existed, and which method was applied to deal with it

*Class imbalance was prevented by preserving the prevalence of positive cases in the training and testing data in the random sampling.*

1. *.* Selection of Machine Learning Models
   1. Explicitly define the goal of the analysis e.g., regression, classification, clustering

*Described under Methods. This study aimed at prediction (classification).*

- 1. Identify the proper learning method used (e.g., supervised, reinforcement learning etc.) to address the problem

*Described in Methods under Machine Learning. Supervised machine learning.*

- 1. Provide explicit details on the use of simpler, complex, or ensemble models

*Described in Methods under Modeling Gradient boosting was used for CCTA and PET data. Final model was built using Cox regression.*

- 1. Provide the comparison of complex models against simpler models if possible

*This report explores the performance of a hybrid approach to event prediction with ML analytics for the integration of imaging results into usable scores that can be modelled through conventional survival analysis.*

- 1. Define ensemble methods, if used

*Described in Methods. Gradient boosting machine was used.*

- 1. Provide details on whether the model is interpretable

*This analysis is contrasted to direct clinical interpretation. We used Cox model to provide more interpretable results of hybridized machine learning scores which are not interpretable on their own.*

1. Model Assessment
   1. Provide a clear description of data used for training, validation, and testing

*Described under Methods. Additionally, the results show the corresponding baseline characteristics.*

- 1. Describe how the model parameters were optimized (e.g., optimization technique, number of model parameters etc.)

*This is described in Methods concerning Machine Learning and in the Supplement under Modeling.*

1. Model Evaluation
   1. Provide the metric(s) used to evaluate the performance of the model

*Performance metrics are clearly described under Statistical Analysis.*

- 1. Define the prevalence of disease and the choice of the scoring rule used

*Documented under Results, Outcomes and survival.*

- 1. Report any methods used to balance the numbers of subjects in each class

*The number of each class was not balanced.*

- 1. Discuss the risk associated to misclassification

*Misclassification risk is, in the case of this particular study, asymmetrical. A false positive result may translate into unnecessary resources and time investment into further analysis, diagnostics and potentially unwarranted treatment. On the other hand, a false negative result may imply more concerning consequences of little to none preventive or therapeutic measures regarding the pathological state.*

1. Best Practices for Model Replicability
   1. Consider sharing code or scripts on a public repository with appropriate copyright protection steps for further development and non-commercial use

*The model structure is available on demand in the format of the generating software utilized for the machine learning analytics.*

- 1. Release a data dictionary with appropriate explanation of the variables

*Vide supra.*

- 1. Document the version of all software and external libraries used

*Described in Methods.*

1. Reporting Limitations, Biases and Alternatives
   1. Identify and report the relevant model assumptions and findings

*Relevant drawbacks are discussed under Limitations.*

- 1. If well performing models were tested on a hold-out validation dataset, detail the data of that validation set with the same rigor as that of training dataset (see section 2 above)

*Cross-validation modeling delivered the testing performance reported. External validation was not performed in this study due to known uncertainties in generalizability under research.*

***Feature Selection***

**Table S1.** Relative influence of CCTA variables in the final ML modelling (GBM).

| RPL atherosis | 45.60 |
| --- | --- |
| LADB atherosis | 8.84 |
| RCAA atherosis | 5.01 |
| LOM1 atherosis | 3.95 |
| RPD calcification | 3.83 |
| D2 calcification | 3.78 |
| RPD atherosis | 2.89 |
| LM calcification | 2.56 |
| LADC calcification | 2.48 |
| RCAB atherosis | 2.43 |
| LCXA atherosis | 2.40 |
| D2 atherosis | 2.17 |
| RCAB calcification | 2.08 |
| LOM2 atherosis | 1.95 |
| LADA atherosis | 1.79 |
| LM atherosis | 1.21 |
| LCXB calcification | 0.99 |
| IM atherosis | 0.93 |
| D1 calcification | 0.88 |
| LCXB atherosis | 0.82 |
| LCXC calcification | 0.76 |
| LADA calcification | 0.57 |
| RCAA calcification | 0.49 |
| LOM1 calcification | 0.48 |
| LCXC atherosis | 0.30 |
| LCXA calcification | 0.30 |
| LADB calcification | 0.28 |
| IM calcification | 0.25 |

**Table S2.** Relative influence of PET variables in the ML model (GBM).

| SEGMENT_13 | 20.87 |
| --- | --- |
| SEGMENT_10 | 20.20 |
| SEGMENT_15 | 8.74 |
| SEGMENT_11 | 7.52 |
| SEGMENT_6 | 6.57 |
| SEGMENT_17 | 6.23 |
| SEGMENT_12 | 6.10 |
| SEGMENT_14 | 5.91 |
| SEGMENT_9 | 4.42 |
| SEGMENT_4 | 3.44 |
| SEGMENT_7 | 2.87 |
| SEGMENT_8 | 2.84 |
| SEGMENT_1 | 1.74 |
| SEGMENT_5 | 1.53 |
| SEGMENT_16 | 1.02 |

***Imaging data***

**Table S3.** Characteristics of CCTA variables.

|  | Training data | | Test data | |  |
| --- | --- | --- | --- | --- | --- |
| Variable | Mean / Number | SD / % | Mean / Number | SD / % | *p*-value |
| Dominance right | 438 | 88.8 | 55 | 88.8 | 0.927 |
| LM atherosis | 1.25 | 0.45 | 1.28 | 0.52 | 0.668 |
| LM stenosis | 1.25 | 0.45 | 1.28 | 0.52 | 0.668 |
| LM calcification | 1.51 | 0.95 | 1.56 | 1.02 | 0.629 |
| LADA atherosis | 1.66 | 0.69 | 1.67 | 0.72 | 0.893 |
| LADA stenosis | 1.69 | 0.78 | 1.68 | 0.74 | 0.938 |
| LADA calcification | 2.16 | 1.18 | 2.15 | 1.18 | 0.945 |
| LADB atherosis | 1.52 | 0.70 | 1.57 | 0.75 | 0.465 |
| LADB stenosis | 1.55 | 0.81 | 1.61 | 0.85 | 0.461 |
| LADB calcification | 1.84 | 1.12 | 1.90 | 1.17 | 0.521 |
| LADC atherosis | 1.21 | 0.49 | 1.22 | 0.52 | 0.869 |
| LADC stenosis | 1.22 | 0.52 | 1.22 | 0.52 | 0.863 |
| LADC calcification | 1.37 | 0.84 | 1.37 | 0.85 | 0.852 |
| D1 atherosis | 1.17 | 0.46 | 1.17 | 0.50 | 0.733 |
| D1 stenosis | 1.17 | 0.49 | 1.17 | 0.52 | 0.731 |
| D1 calcification | 1.30 | 0.81 | 1.27 | 0.76 | 0.675 |
| D2 atherosis | 0.79 | 0.60 | 0.80 | 0.62 | 0.932 |
| D2 stenosis | 0.79 | 0.62 | 0.80 | 0.62 | 0.935 |
| D2 calcification | 0.83 | 0.74 | 0.85 | 0.76 | 0.905 |
| LCXA atherosis | 1.36 | 0.60 | 1.32 | 0.61 | 0.186 |
| LCXA stenosis | 1.36 | 0.62 | 1.34 | 0.69 | 0.193 |
| LCXA calcification | 1.63 | 1.04 | 1.54 | 0.99 | 0.179 |
| LCXB atherosis | 1.18 | 0.46 | 1.2 | 0.51 | 0.758 |
| LCXB stenosis | 1.19 | 0.52 | 1.22 | 0.6 | 0.753 |
| LCXB calcification | 1.31 | 0.79 | 1.33 | 0.81 | 0.803 |
| LCXC atherosis | 1.09 | 0.32 | 1.09 | 0.35 | 0.952 |
| LCXC stenosis | 1.09 | 0.32 | 1.09 | 0.35 | 0.952 |
| LCXC calcification | 1.16 | 0.58 | 1.17 | 0.63 | 0.889 |
| LPD atherosis | 1.63 | 1.04 | 1.54 | 0.99 | 0.805 |
| LPD stenosis | 1.18 | 0.46 | 1.20 | 0.51 | 0.805 |
| LPD calcification | 1.19 | 0.52 | 1.22 | 0.6 | 0.805 |
| LOM1 atherosis | 1.31 | 0.79 | 1.33 | 0.81 | 0.993 |
| LOM1 stenosis | 1.09 | 0.32 | 1.09 | 0.35 | 0.993 |
| LOM1 calcification | 1.09 | 0.32 | 1.09 | 0.35 | 0.952 |
| LOM2 atherosis | 1.16 | 0.58 | 1.17 | 0.63 | 0.472 |
| LOM2 stenosis | 0.14 | 0.39 | 0.15 | 0.38 | 0.472 |
| LOM2 calcification | 0.14 | 0.39 | 0.15 | 0.38 | 0.420 |
| IM atherosis | 0.15 | 0.42 | 0.15 | 0.42 | 0.802 |
| IM stenosis | 1.08 | 0.33 | 1.08 | 0.31 | 0.803 |
| IM calcification | 1.08 | 0.33 | 1.08 | 0.31 | 0.801 |
| LPL atherosis | 1.14 | 0.55 | 1.15 | 0.59 | 0.379 |
| LPL stenosis | 0.16 | 0.42 | 0.17 | 0.39 | 0.379 |
| LPL calcification | 0.16 | 0.42 | 0.17 | 0.39 | 0.380 |
| RCAA atherosis | 0.18 | 0.51 | 0.17 | 0.41 | 0.636 |
| RCAA stenosis | 0.31 | 0.66 | 0.32 | 0.65 | 0.653 |
| RCAA calcification | 0.32 | 0.67 | 0.32 | 0.65 | 0.826 |
| RCAB atherosis | 0.38 | 0.87 | 0.37 | 0.84 | 0.871 |
| RCAB stenosis | 0.25 | 0.50 | 0.20 | 0.42 | 0.874 |
| RCAB calcification | 0.25 | 0.50 | 0.20 | 0.42 | 0.996 |
| RCAC atherosis | 0.26 | 0.56 | 0.21 | 0.47 | 0.920 |
| RCAC stenosis | 1.43 | 0.66 | 1.41 | 0.66 | 0.924 |
| RCAC calcification | 1.46 | 0.74 | 1.45 | 0.78 | 0.998 |
| RPD atherosis | 1.75 | 1.10 | 1.74 | 1.14 | 0.845 |
| RPD stenosis | 1.30 | 0.58 | 1.30 | 0.59 | 0.845 |
| RPD calcification | 1.32 | 0.66 | 1.33 | 0.69 | 0.565 |
| RPL atherosis | 1.02 | 0.31 | 1.03 | 0.33 | 0.696 |
| RPL stenosis | 1.06 | 0.48 | 1.09 | 0.61 | 0.696 |
| RPL calcification | 1.25 | 0.45 | 1.28 | 0.52 | 0.971 |

Atherosis values are 0: No segment (the patient does not have this particular segment in his/her coronaries), 1: Normal segment, 2: Non-significant plaque and 3: Significant Plaque.

Stenosis values are 0: No stenosis in the segment because the segment does not exist, 1: Normal segment (no stenosis in a patient who has the segment, but it is normal), 2: <50% stenosis, 3: 50-67% stenosis, 4: 70-99% stenosis and 5: 100% stenosis (occlusion).

Calcification values are 0: Non-existing segment (the patient does not have this particular segment in his/her coronaries), 1: Normal segment (the patient has the segment, but it is normal), 2: Soft plaque (non-calcified plaque), 3: Calcified plaque and 4: Mixed plaque.

**Table S4.** Characteristics of PET variables.

|  | Training data | | Test data | |  |
| --- | --- | --- | --- | --- | --- |
| Variable | Mean | Sd | Mean | Sd | p-values |
| SEGMENT_1 | 1.37 | 1.62 | 1.42 | 1.69 | 0.840 |
| SEGMENT_4 | 1.47 | 1.84 | 1.48 | 1.81 | 0.808 |
| SEGMENT_5 | 1.48 | 1.78 | 1.53 | 1.78 | 0.664 |
| SEGMENT_6 | 1.49 | 1.76 | 1.49 | 1.72 | 0.918 |
| SEGMENT_7 | 1.46 | 1.73 | 1.51 | 1.76 | 0.823 |
| SEGMENT_8 | 1.34 | 1.62 | 1.40 | 1.68 | 0.738 |
| SEGMENT_9 | 1.35 | 1.66 | 1.40 | 1.67 | 0.650 |
| SEGMENT_10 | 1.49 | 1.83 | 1.53 | 1.80 | 0.743 |
| SEGMENT_11 | 1.65 | 2.01 | 1.66 | 1.94 | 0.768 |
| SEGMENT_12 | 1.57 | 1.83 | 1.56 | 1.78 | 0.892 |
| SEGMENT_13 | 1.53 | 1.84 | 1.57 | 1.89 | 0.816 |
| SEGMENT_14 | 1.45 | 1.82 | 1.48 | 1.76 | 0.684 |
| SEGMENT_15 | 1.62 | 2.04 | 1.60 | 1.88 | 0.851 |
| SEGMENT_16 | 1.70 | 2.02 | 1.70 | 1.97 | 0.936 |
| SEGMENT_17 | 1.70 | 2.08 | 1.70 | 1.98 | 0.850 |

***Clinical data***

**Table S5.** Missing values as percentages in training and testing data.

| Variable | Training Data | Test Data |
| --- | --- | --- |
| Smoking status | 18.5% | 11.4% |
| Type 2 Diabetes | 23.5% | 19.1% |
| Hypertension | 16.2% | 17.5% |
| Dyslipidemia | 17.2% | 16.3% |

***ML modelling***

We built ML regression models using the training dataset and generalized boosted regression (GBM)^6^. We utilized 10-fold cross-validation and default number of 100 trees. The modelling was done using R (version 3.5.3) and the following R packages: survival (version 2.44-1.1) and survcomp (version 1.36.1) to implement Cox models, and gbm (version 2.1.5) for GBM. The regression values from ML models were used together with clinical variables to build Cox regression model.
