# Supplementary material for: Hybridizing machine learning in survival analysis of cardiac PET/CT imaging
Source: J Nucl Cardiol. 2023 Sep 1;30(6):2750–9. doi: 10.1007/s12350-023-03359-4 (PMC10682215; doi:10.1007/s12350-023-03359-4)
Supplement: Supplementary file 2 — Supplementary file2 (PPTX 1170 kb) [file 12350_2023_3359_MOESM2_ESM.pptx]

## Slide 1
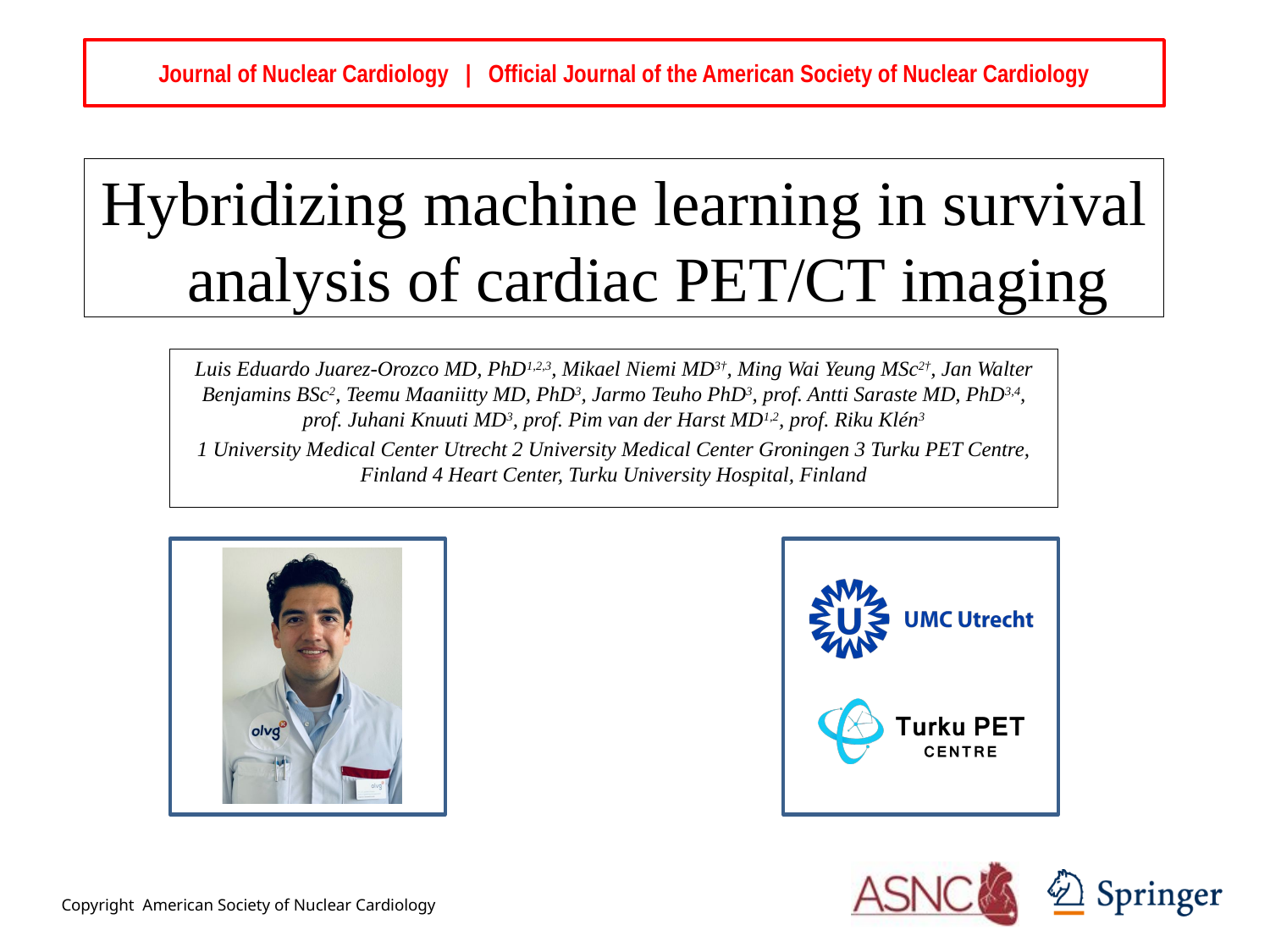

Journal of Nuclear Cardiology | Official Journal of the American Society of Nuclear Cardiology
# Hybridizing machine learning in survival analysis of cardiac PET/CT imaging
Luis Eduardo Juarez-Orozco MD, PhD1,2,3, Mikael Niemi MD3†, Ming Wai Yeung MSc2†, Jan Walter Benjamins BSc2, Teemu Maaniitty MD, PhD3, Jarmo Teuho PhD3, prof. Antti Saraste MD, PhD3,4, prof. Juhani Knuuti MD3, prof. Pim van der Harst MD1,2, prof. Riku Klén3
1 University Medical Center Utrecht 2 University Medical Center Groningen 3 Turku PET Centre, Finland 4 Heart Center, Turku University Hospital, Finland
Copyright American Society of Nuclear Cardiology

## Slide 2
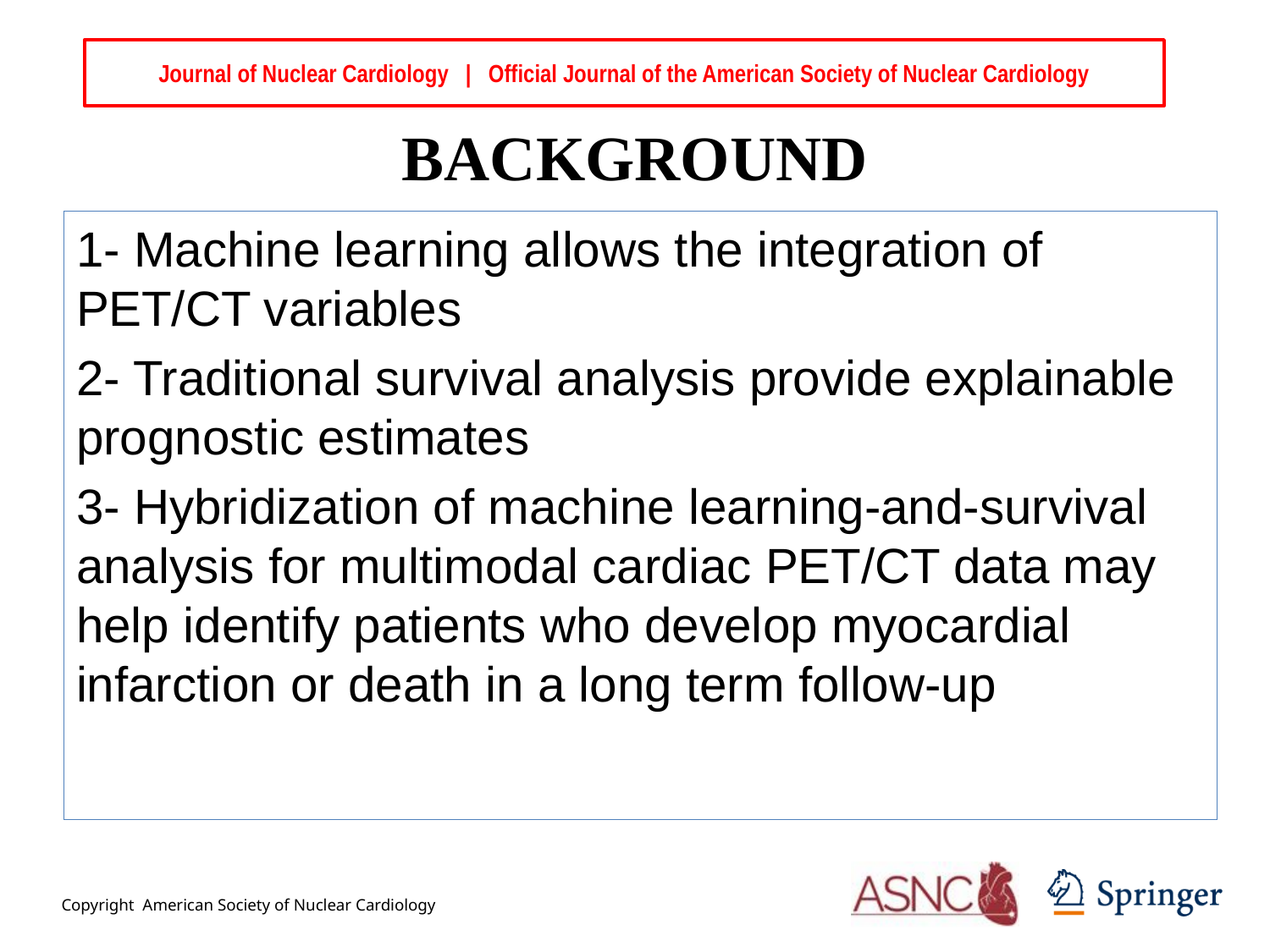

Journal of Nuclear Cardiology | Official Journal of the American Society of Nuclear Cardiology
# BACKGROUND
1- Machine learning allows the integration of PET/CT variables
2- Traditional survival analysis provide explainable prognostic estimates
3- Hybridization of machine learning-and-survival analysis for multimodal cardiac PET/CT data may help identify patients who develop myocardial infarction or death in a long term follow-up
Copyright American Society of Nuclear Cardiology

## Slide 3
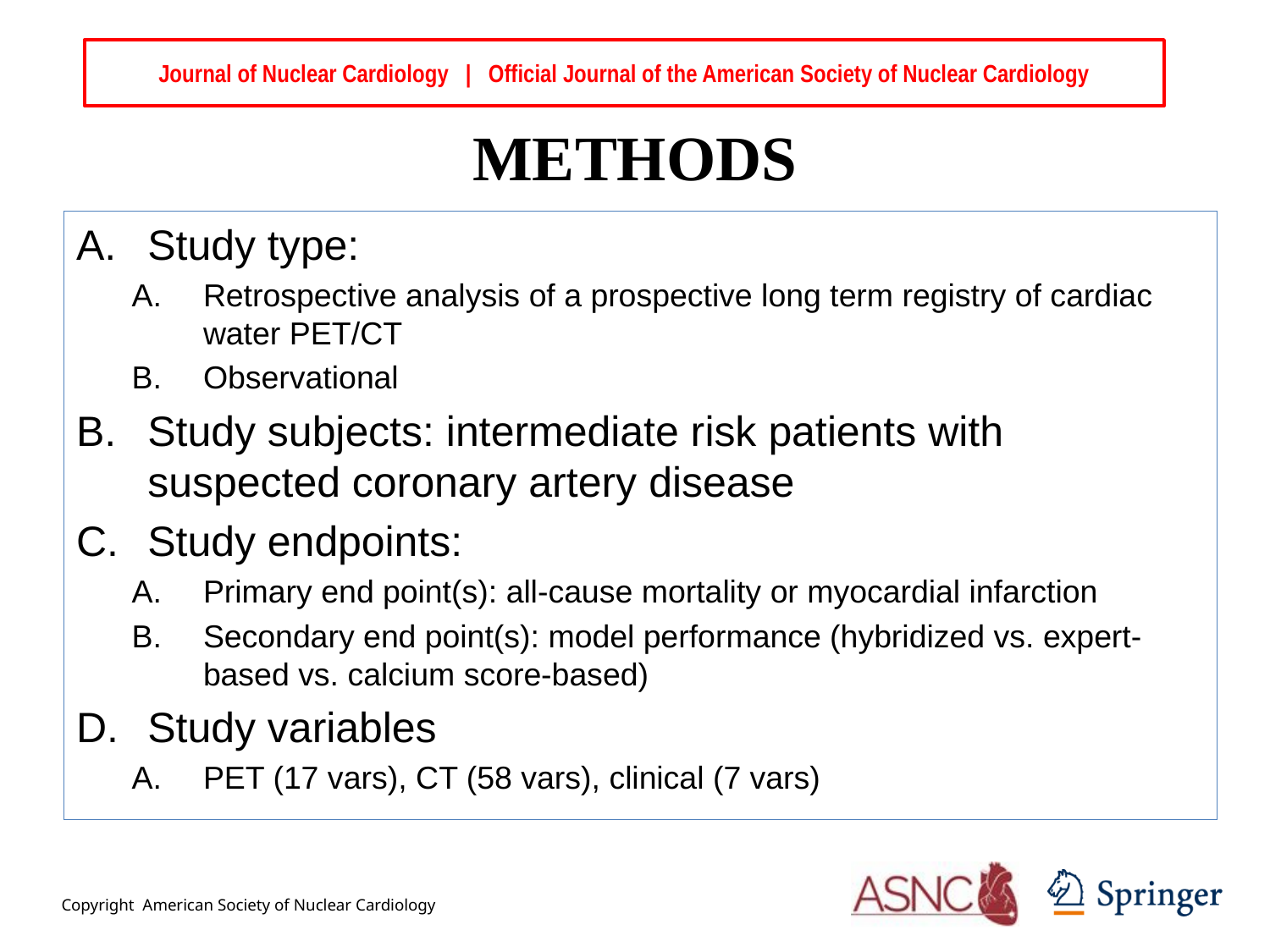

Journal of Nuclear Cardiology | Official Journal of the American Society of Nuclear Cardiology
# METHODS
Study type:
Retrospective analysis of a prospective long term registry of cardiac water PET/CT
Observational
Study subjects: intermediate risk patients with suspected coronary artery disease
Study endpoints:
Primary end point(s): all-cause mortality or myocardial infarction
Secondary end point(s): model performance (hybridized vs. expert-based vs. calcium score-based)
Study variables
PET (17 vars), CT (58 vars), clinical (7 vars)
Copyright American Society of Nuclear Cardiology

## Slide 4
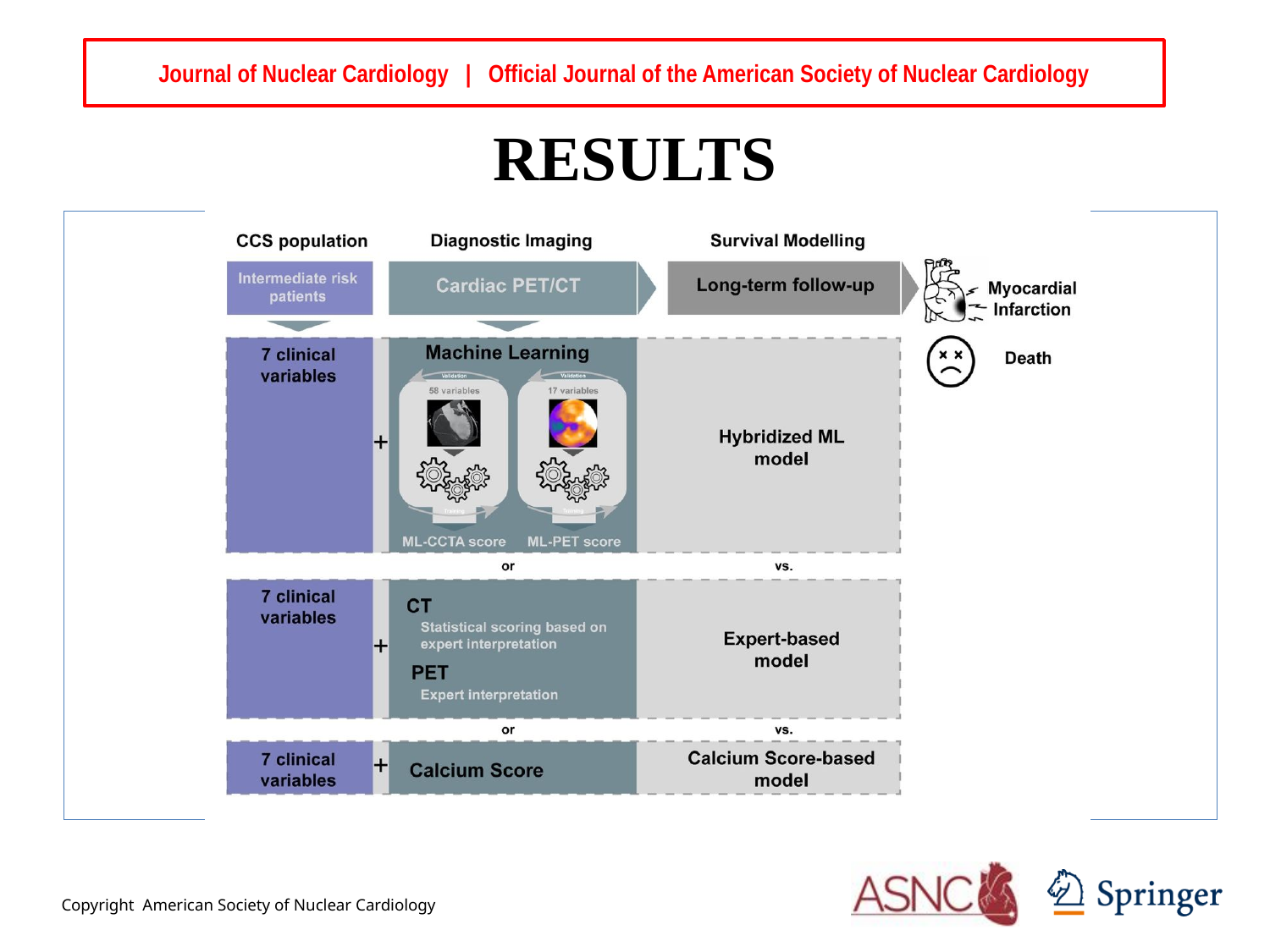

Journal of Nuclear Cardiology | Official Journal of the American Society of Nuclear Cardiology
# RESULTS
Copyright American Society of Nuclear Cardiology

## Slide 5
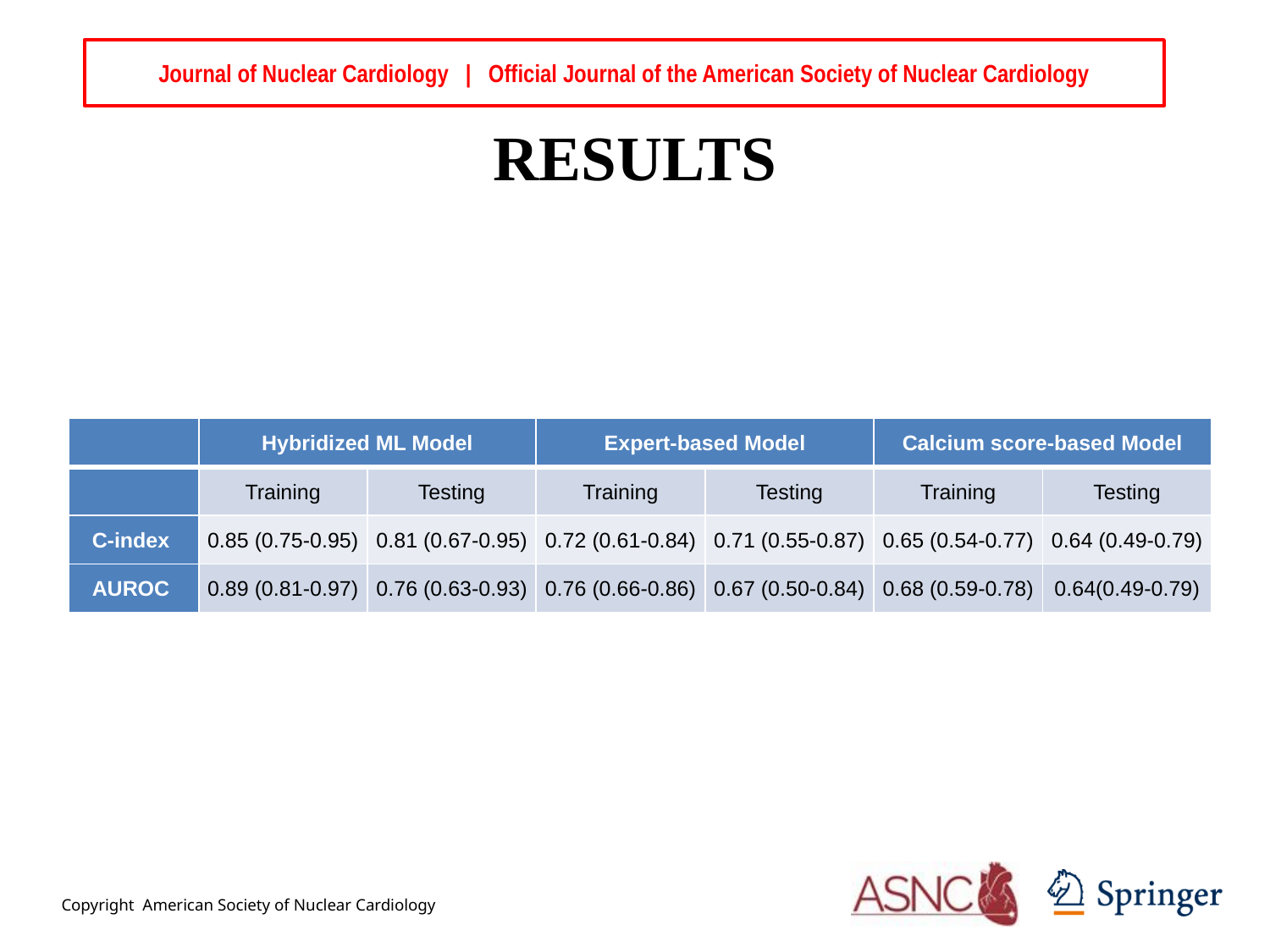

Journal of Nuclear Cardiology | Official Journal of the American Society of Nuclear Cardiology
# RESULTS
| | Hybridized ML Model | | Expert-based Model | | Calcium score-based Model | |
| --- | --- | --- | --- | --- | --- | --- |
| | Training | Testing | Training | Testing | Training | Testing |
| C-index | 0.85 (0.75-0.95) | 0.81 (0.67-0.95) | 0.72 (0.61-0.84) | 0.71 (0.55-0.87) | 0.65 (0.54-0.77) | 0.64 (0.49-0.79) |
| AUROC | 0.89 (0.81-0.97) | 0.76 (0.63-0.93) | 0.76 (0.66-0.86) | 0.67 (0.50-0.84) | 0.68 (0.59-0.78) | 0.64(0.49-0.79) |
Copyright American Society of Nuclear Cardiology

## Slide 6
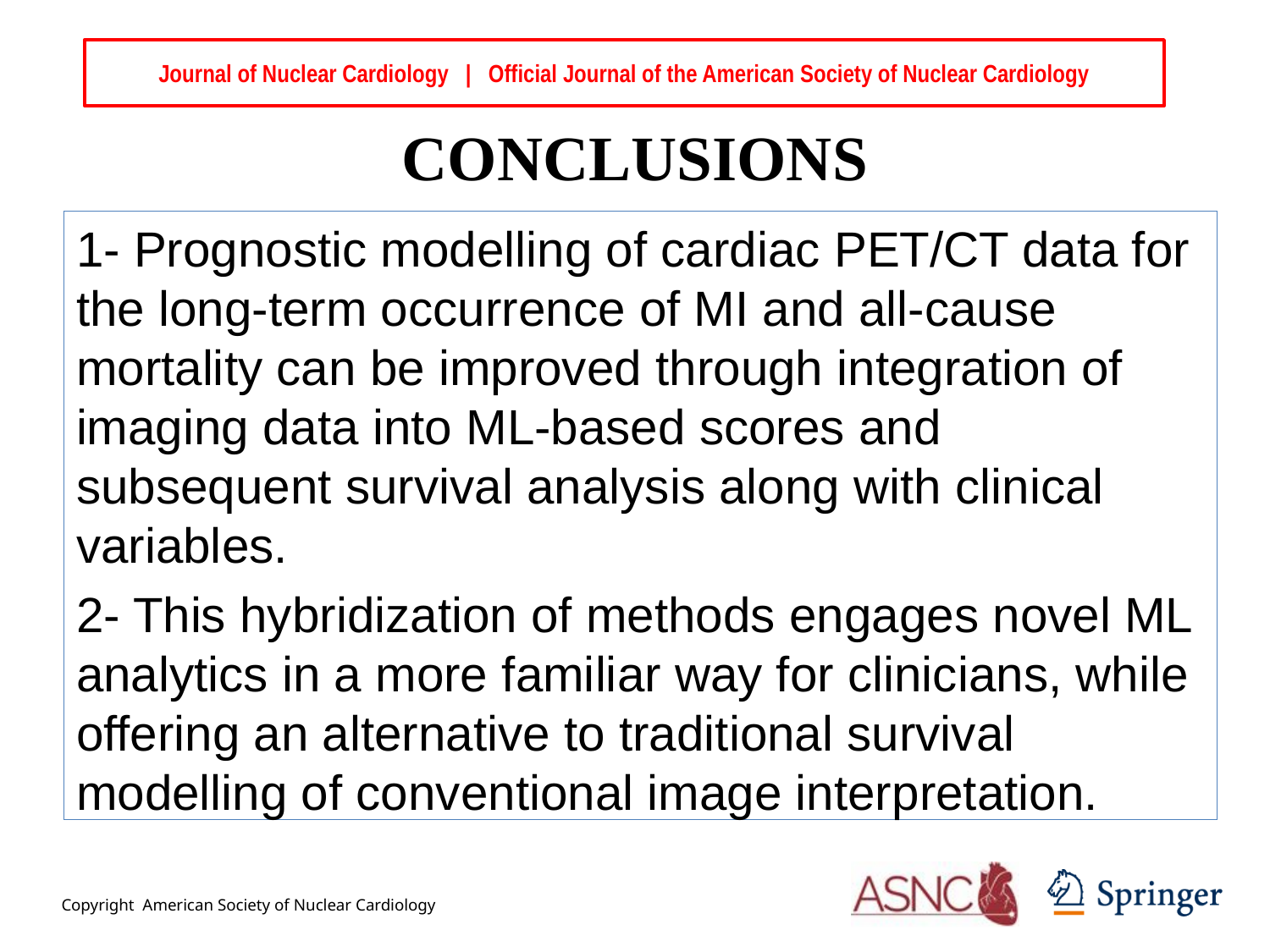

Journal of Nuclear Cardiology | Official Journal of the American Society of Nuclear Cardiology
# CONCLUSIONS
1- Prognostic modelling of cardiac PET/CT data for the long-term occurrence of MI and all-cause mortality can be improved through integration of imaging data into ML-based scores and subsequent survival analysis along with clinical variables.
2- This hybridization of methods engages novel ML analytics in a more familiar way for clinicians, while offering an alternative to traditional survival modelling of conventional image interpretation.
Copyright American Society of Nuclear Cardiology
